# Infertility, anxiety, and depression among adolescents and young adults with cancer: the Mexico Cancer Survivorship Registry

**DOI:** 10.1093/oncolo/oyag062

**Published:** 2026-03-09

**Authors:** Heidy N Medina, Patricia I Moreno, Frank J Penedo, Johis Ortega, Erika Ruiz-García, Pasquale Patrizio, Oscar Galindo Vázquez

**Affiliations:** Sylvester Comprehensive Cancer Center, University of Miami Miller School of Medicine, Miami, FL, 33136, United States; School of Nursing and Health Studies, University of Miami, Coral Gables, FL, 33146, United States; Sylvester Comprehensive Cancer Center, University of Miami Miller School of Medicine, Miami, FL, 33136, United States; Department of Public Health Sciences, University of Miami Miller School of Medicine, Miami, FL, 33136, United States; Sylvester Comprehensive Cancer Center, University of Miami Miller School of Medicine, Miami, FL, 33136, United States; Department of Psychology, University of Miami, Coral Gables, FL, 33146, United States; School of Nursing and Health Studies, University of Miami, Coral Gables, FL, 33146, United States; Translational Medicine Laboratory and Department of Gastrointestinal Tumors, Instituto Nacional de Cancerología, Mexico City, 14080, Mexico; Department of Obstetrics, Gynecology, and Reproductive Sciences, University of Miami School of Medicine, Miami, FL, 33136, United States; Department of Mental Health, Instituto Nacional de Cancerología, Mexico City, 14080, Mexico

**Keywords:** adolescents and young adults, cancer survivor, infertility, distress, anxiety, depression, Mexico

## Abstract

**Background:**

In 2020, 24,000 new cancer cases were diagnosed among adolescents and young adults (AYAs) in Mexico. Cancer-related infertility affects 30%-75% of AYAs and is associated with poor quality of life, relationship satisfaction, and self-worth. This study examines the association between infertility, anxiety, and depression among AYA cancer survivors in Mexico.

**Methods:**

Data for AYAs (ages 15-39) in the Registro de Supervivientes de Cancer (Cancer Survivor Registry) developed by the Instituto Nacional de Cancerología in Mexico was utilized. A self-report survey was conducted during 2014-2018. Logistic regression models were used to calculate odds ratios (ORs) and corresponding 95% confidence intervals (CIs).

**Results:**

AYA cancer survivors (*N* = 1168) had a median age of 31 (interquartile range: 25-36), were predominantly women (75%), and 42% had a college education or higher. The most common cancers were breast (33%), lymphoma (12%), cervical (10%), and testicular (9%) with the majority being Stage III (19%) tumors. Approximately 1 in 8 AYAs (12%) reported infertility. Across men and women, after adjusting for age at diagnosis, time since the end of treatment, education level, geographical region, stage, treatment type, and cancer type, AYAs who reported infertility were more likely to experience depression (OR 1.52, 95% CI: 1.02-2.26) symptoms than those who did not report infertility. There was no association between infertility and anxiety among all AYA cancer survivors combined.

**Conclusions:**

Infertility is associated with depression symptoms among AYA Mexican cancer survivors. Mexican AYA cancer survivors experiencing infertility may need additional support to address unmet care needs.

Implications for Practice There is a significant gap in research on the experiences of fertility among men and women with different cancer types in Mexico. We analyzed data from the Registro de Supervivientes de Cancer (Cancer Survivor Registry) developed by the Instituto Nacional de Cancerología in Mexico, a comprehensive yet underutilized database. This survey included a large diverse nationwide sample of cancer survivors. Our findings indicate that across AYAs, those who reported infertility were more likely to experience depression symptoms. Our study highlights the urgent need for additional support and infrastructure to address the unmet care needs of AYA Mexican cancer survivors experiencing infertility.

## Introduction

Although advancements in early detection and cancer therapies have substantially enhanced survival rates among cancer survivors, many life-preserving treatments can exert iatrogenic effects on reproductive endocrine function and fertility.^1^ Gonadotoxic therapies including chemotherapy (ie, alkylating agents) and radiation therapy as well as surgery, can either directly or indirectly damage reproductive organs resulting in reproductive difficulties.^2^ In 2006, the term “oncofertility” was created to describe the merging of two fields, oncology and reproductive health.^3^ A number of well-established fertility preservation options exist for women and men including oocyte or embryo cryopreservation, ovarian cortical tissue cryopreservation, and sperm or testicular issue cryopreservation before the initiation of cancer-directed therapy.^1^ Infertility and impaired reproductive function are critically important among adolescent and young adult (AYA) cancer survivors, with nearly 45% reporting that having children is among their top three life goals.^4^ Infertility among AYAs varies widely, from 13% up to 75%, depending on sex, cancer type, treatment, follow-up duration, and how infertility is defined and measured, among other factors.^4^^,^^5^

Reproductive concerns impact quality of life and psychological well-being and infertility increases symptoms of depression and anxiety and decreases self-worth and life and relationship satisfaction among AYA cancer survivors.^4^^,^^6–8^ The potential risk of infertility is associated with a sense of loss and anger,^9^ influencing an individual’s acceptance of their cancer diagnosis,^10^ given that preserving the possibility of having children after cancer, in many cases, may serve as a stimulus for recovery.^11^ A higher prevalence of infertility-related distress among female than male AYAs has been observed, possibly due to higher unmet informational needs among women with cancer^4^ given that they are less likely to be offered fertility preservation counseling or referral to a specialist.^12^^,^^13^

In 2020, there were nearly 24,000 new cases of cancer diagnosed among AYA men and women in Mexico, representing 12% of all new cases.^14^ In resource-limited countries such as Mexico, the adoption and implementation of oncofertility practices are limited.^15^ Cancer control efforts primarily prioritize treatment over survivorship care, with limited availability and planning for fertility preservation.^16^ Although fertility-preserving technologies are often available,^17^ preservation strategies are rarely covered by private or government insurance and therefore inaccessible to underserved populations.^18^ A recent survey among patients with breast cancer in Mexico demonstrated that 44% reported fertility preservation concerns, yet only 3% could afford fertility preservation.^17^ Additionally, health system and provider barriers include a lack of knowledge about the safety of preservation methods and pregnancy after a cancer diagnosis, a shortage of reproductive and fertility specialists, and a lack of partnerships between oncology teams and reproductive units.^19^^,^^20^ Patient knowledge regarding preservation options is also low.^17^ From a cultural perspective, conservative religious, cultural, and ethical attitudes in Mexico also play a role in the acceptability of fertility preservation.^17^

Currently, there is a paucity of research on experiences of fertility among men and women of diverse cancer types, specifically in low-resource settings. Therefore, the primary aim of this study was to assess the relationship between infertility, anxiety, and depression among AYA cancer survivors in Mexico.

## Methods

### Data source and population

Data for cancer survivors who participated in the Registro de Supervivientes de Cancer (Cancer Survivor Registry) developed by the Instituto Nacional de Cancerología (INCAN, National Cancer Institute of Mexico) and INFOCANCER, an information center for patients with cancer, their family members, and the general public, in Mexico was utilized. Prior to deployment, the content validity of this self-report survey was assessed by conducting a focus group with nine health professionals. The survey was then pilot-tested with ten cancer survivors. After incorporating feedback, the self-report survey was administered online via convenience sampling from 2014 to 2018 to understand the physical, emotional, and social needs of cancer survivors in Mexico. Any individual 18 years of age or older at the time of survey completion, with a medical history of a cancer diagnosis, and currently in the post-treatment phase of the cancer continuum was eligible to participate. This registry consists of a total sample of 3201 cancer survivors across Mexico who had a median age of 55 years old and were predominantly patients at INCAN (∼71%), women (∼83%), and 79% were 5 or more years post-treatment completion. Among this entire sample, 97% reported cancer-related side effects and 79% reported psychosocial alterations. We extracted data for AYA cancer survivors (ie, individuals diagnosed with cancer at ages 15-39), specifically, which comprised 37% of the total sample (*n* = 1168).

Information was collected about sociodemographic and clinical characteristics as well as secondary effects. Sociodemographic data regarding geographical region of residence, sex, date of birth (age), occupation, and level of education were collected. Information on secondary effects, including anxiety and depression and physical symptoms was also assessed. Items include (*Yes* or *No*): “Have you experienced the following side effects?…Infertility (ie, inability to achieve pregnancy)?” and “Have you experienced the following psychosocial problems?…Depression?…Anxiety?” (Tables S1 and S2). Additionally, clinically relevant variables regarding tumor site, date of diagnosis, date of treatment initiation and termination, date of data capture for registry, cancer stage at diagnosis, hospital where treatment was attained, and type of treatment received (surgery, chemotherapy, radiotherapy, brachytherapy, hormone therapy, monoclonal antibodies, radioactive iodine) were evaluated.

### Data analyses

We assessed the frequencies for all sociodemographic and tumor-related characteristics and how often anxiety and depression were reported for AYA cancer survivors in Mexico and used chi-square tests to compare differences in frequency distributions in males versus females. We used separate logistic regression models to evaluate the relationship of self-reported infertility (Yes vs No) with anxiety and with depression, modeling the presence of each symptom and estimating odds ratios (ORs) with 95% confidence intervals (CIs). We ran univariable (ie, without adjustment for additional variables) models followed by multivariable models adjusted for age at diagnosis, time since the end of treatment, sex, education level, geographical region of residence, tumor site, cancer stage, and receipt of surgery, chemotherapy, radiotherapy, and hormone therapy. We report results for males and females separately, and for the combined sample. We conducted sensitivity analyses restricted to individuals diagnosed at 18 years or older. Analyses were conducted using SAS v9.4 software (SAS Institute, Cary, NC, USA). All statistical tests were 2-sided with a statistical significance of *P* < .05.

## Results

### AYA cancer survivor sociodemographic and clinical characteristics and anxiety and depression

From 2014 to 2018, AYA cancer survivors in Mexico (*N* = 1168, Table 1) had a median age at diagnosis of 31 (interquartile range: 25-36), were predominantly 5 to 9 years post-treatment (30%), women (75%), 42% had a college education or higher, and 40% resided in Mexico City. The most common tumor sites among all AYAs were breast (33%), lymphoma (12%), cervical (10%), and testicular (9%) cancer with the majority being Stage III (19%) tumors, and 12% of AYAs reported post-treatment infertility. Overall, 42% and 46% of AYAs reported having symptoms of anxiety and depression, respectively. By sex, women were more likely to be 10 years or more in the post-treatment phase (29%), while men were more commonly 5 to 9 years post-treatment (34%). Women had a higher proportion of having less than a high school education than males (38% vs 19%), while men had a higher proportion of college education or higher than women (53% vs 38%). AYA women were more likely to report anxiety (46%) and depression (50%) than men (30% and 32%, respectively).

**Table 1:** Sociodemographic and clinical characteristics and anxiety and depression among adolescents and young adults with cancer in Mexico, 2014-2018.

	Females (*N* = 881, 75.4%)	Males (*N* = 287, 24.6%)	**Total** **(*N* = 1168, 100.0%)**	*P*-value^a^
**Median age at diagnosis (years)**	33	27	31	
**Median time since end of treatment (years)**	6	6	6	
**Age at diagnosis (years)**				<.0001
**15-17**	37 (4.2%)	22 (7.7%)	59 (5.1%)	
**18-25**	141 (16.0%)	101 (35.2%)	242 (20.7%)	
**26-39**	703 (79.8%)	164 (57.1%)	867 (74.2%)	
**Time since end of treatment (years)**				.16
**<1**	141 (16.0%)	51 (17.8%)	192 (16.4%)	
**1-4**	235 (26.7%)	63 (22.0%)	298 (25.5%)	
**5-9**	248 (28.2%)	97 (33.8%)	345 (29.5%)	
**≥10**	257 (29.2%)	76 (26.5%)	333 (28.5%)	
**Education level**				<.0001
**Less than high school**	332 (37.7%)	54 (18.8%)	386 (33.1%)	
**High school**	196 (22.3%)	73 (25.4%)	269 (23.0%)	
**College or higher**	336 (38.1%)	151 (52.6%)	487 (41.7%)	
**Unknown**	17 (1.9%)	9 (3.1%)	26 (2.2%)	
**Region**				.16
**Northwest**	32 (3.6%)	8 (2.8%)	40 (3.4%)	
**Northeast**	214 (24.3%)	89 (31.0%)	303 (25.9%)	
**West and Lowlands**	74 (8.4%)	25 (8.7%)	99 (8.5%)	
**Mexico City**	368 (41.8%)	98 (34.2%)	466 (39.9%)	
**South Central and East**	126 (14.3%)	41 (14.3%)	167 (14.3%)	
**South**	67 (7.6%)	26 (9.1%)	93 (8.0%)	
**Infertility**				.07
**Yes**	112 (12.7%)	25 (8.7%)	137 (11.7%)	
**No**	769 (87.3%)	262 (91.3%)	1031 (88.3%)	
**Tumor sites^b^**				<.0001
**Breast**	375 (42.6%)	4 (1.4%)	379 (32.5%)	
**Testicular**	—	106 (36.9%)	106 (9.1%)	
**Ovarian**	62 (7.0%)	—	62 (5.3%)	
**Cervical**	117 (13.3%)	—	117 (10.0%)	
**Colorectal**	27 (3.1%)	26 (9.1%)	53 (4.5%)	
**Head and neck**	26 (3.0%)	9 (3.1%)	35 (3.0%)	
**Thyroid**	53 (6.0%)	8 (2.8%)	61 (5.2%)	
**Lymphoma**	72 (8.2%)	72 (25.1%)	144 (12.3%)	
**Leukemia**	29 (3.3%)	13 (4.5%)	42 (3.6%)	
**Other**	120 (13.6%)	49 (17.1%)	169 (14.5%)	
**Cancer stage**				.40
**Stage 0**	53 (6.0%)	21 (7.3%)	74 (6.3%)	
**Stage I**	126 (14.3%)	50 (17.4%)	176 (15.1%)	
**Stage II**	161 (18.3%)	42 (14.6%)	203 (17.4%)	
**Stage III**	173 (19.6%)	49 (17.1%)	222 (19.0%)	
**Stage IV**	74 (8.4%)	21 (7.3%)	95 (8.1%)	
**Unknown**	294 (33.4%)	104 (36.2%)	398 (34.1%)	
**Surgery**				<.0001
**Yes**	739 (83.9%)	205 (71.4%)	944 (80.8%)	
**No**	142 (16.1%)	82 (28.6%)	224 (19.2%)	
**Chemotherapy**				.02
**Yes**	636 (72.2%)	228 (79.4%)	864 (74.0%)	
**No**	245 (27.8%)	59 (20.6%)	304 (26.0%)	
**Radiotherapy**				<.0001
**Yes**	429 (48.7%)	86 (30.0%)	515 (44.1%)	
**No**	452 (51.3%)	201 (70.0%)	653 (55.9%)	
**Hormone therapy**				<.0001
**Yes**	181 (20.5%)	3 (1.1%)	184 (15.8%)	
**No**	700 (79.5%)	284 (99.0%)	984 (84.3%)	
**Anxiety**				<.0001
**Yes**	405 (46.0%)	87 (30.3%)	492 (42.1%)	
**No**	476 (54.0%)	200 (69.7%)	676 (57.9%)	
**Depression**				<.0001
**Yes**	443 (50.3%)	92 (32.1%)	535 (45.8%)	
**No**	438 (49.7%)	195 (67.9%)	633 (54.2%)	

a *P*-values were calculated using the chi-square test or Fisher’s exact test, as appropriate, to assess differences in proportions between males and females.

b Most common cancer types observed in this population, all others included as “other.”

### Associations of reported infertility with anxiety symptoms

For all AYA cancer survivors in Mexico combined, in univariable analysis, when not adjusting for other variables, those who reported infertility were more likely to have anxiety symptoms (OR 1.56, 95% CI: 1.09-2.23) than those who did not report infertility. In multivariable analysis, after adjusting for age at diagnosis, time since end of treatment, education level, region, cancer treatment received (Table 2, Total, Model 1) as well as further adjustment for cancer type (Table 2, Total, Model 2), there were no associations between reported infertility and anxiety. By sex, women who reported infertility were more likely than women who did not report infertility to have anxiety symptoms with (Table 2, Female, Model 1, OR 1.50, 95% CI: 0.99-2.28) and without adjustment (OR 1.54, 95% CI: 1.03-2.30). However, this association was no longer present after adjusting for cancer type as well (Table 2, Female, Model 2). Women with breast cancer (Table 2, Female, Model 2, OR 0.43, 95% CI: 0.21-0.88) and lymphoma (Table 2, Female, Model 2, OR 0.39, 95% CI: 0.16-0.93) were less likely to endorse anxiety symptoms than women with thyroid cancer. Among men, infertility was not associated with anxiety in univariable (OR 1.33, 95% CI: 0.56-3.13) or multivariable (Table 2, Male, Models 1/2) analyses.

**Table 2:** Predictors of anxiety^a^ symptoms in adolescents and young adults with cancer in Mexico, 2014-2018.

	Female		Male		Total	
	Model 1^b^	Model 2^c^	Model 1^b^	Model 2^c^	Model 1^b^	Model 2^c^
	OR (95% CI)^d^	OR (95% CI)^d^	OR (95% CI)^d^	OR (95% CI)^d^	OR (95% CI)^d^	OR (95% CI)^d^
**Infertility**						
**No**	Ref	Ref	Ref	Ref	Ref	Ref
**Yes**	**1.50 (0.99-2.28)**	1.46 (0.95-2.24)	1.01 (0.37-2.78)	1.05 (0.36-3.02)	1.41 (0.97-2.05)	1.37 (0.93-2.02)
**Sex**						
**Male**	—	—	—	—	Ref	Ref
**Female**	—	—	—	—	**1.73 (1.27-2.37)**	1.48 (0.97-2.24)
**Age at diagnosis (years)**						
**15-17**	Ref	Ref	Ref	Ref	Ref	Ref
**18-25**	0.65 (0.30-1.42)	0.71 (0.32-1.57)	0.59 (0.20-1.79)	0.74 (0.23-2.34)	0.65 (0.35-1.20)	0.70 (0.38-1.30)
**26-39**	0.56 (0.27-1.16)	0.64 (0.30-1.37)	0.87 (0.30-2.49)	1.24 (0.41-3.75)	0.68 (0.38-1.21)	0.78 (0.43-1.41)
**Time since end of treatment (years)**						
**<1**	Ref	Ref	Ref	Ref	Ref	Ref
**1-4**	1.16 (0.74-1.81)	1.18 (0.75-1.86)	0.83 (0.35-1.99)	0.92 (0.36-2.37)	1.15 (0.78-1.70)	1.21 (0.81-1.79)
**5-9**	1.07 (0.68-1.66)	1.08 (0.69-1.70)	0.79 (0.34-1.82)	0.85 (0.34-2.15)	1.06 (0.72-1.55)	1.08 (0.73-1.59)
**≥10**	0.97 (0.62-1.53)	1.02 (0.64-1.61)	0.69 (0.28-1.66)	0.64 (0.25-1.66)	0.96 (0.65-1.42)	1.01 (0.68-1.51)
**Education level**						
**College or higher**	Ref	Ref	Ref	Ref	Ref	Ref
**High school**	**1.66 (1.15-2.41)**	**1.63 (1.12-2.37)**	0.74 (0.38-1.43)	0.70 (0.34-1.41)	1.36 (0.99-1.86)	1.33 (0.96-1.82)
**Less than high school**	**1.44 (1.03-2.02)**	1.40 (0.99-1.99)	0.68 (0.32-1.48)	0.90 (0.39-2.06)	1.22 (0.91-1.65)	1.25 (0.91-1.70)
**Unknown**	0.32 (0.07-1.57)	0.29 (0.06-1.47)	0.36 (0.04-3.22)	0.49 (0.05-4.71)	0.31 (0.09-1.11)	0.29 (0.08-1.08)
**Region**						
**Northeast**	Ref	Ref	Ref	Ref	Ref	Ref
**Northwest**	1.63 (0.71-3.76)	1.88 (0.81-4.37)	—	—	1.06 (0.51-2.23)	1.22 (0.57-2.59)
**West and Lowlands**	**1.76 (1.01-3.07)**	**1.81 (1.03-3.17**)	0.85 (0.29-2.51)	0.79 (0.25-2.50)	1.50 (0.92-2.42)	1.54 (0.95-2.51)
**Mexico City**	1.28 (0.90-1.82)	1.27 (0.89-1.81)	1.14 (0.57-2.28)	1.12 (0.54-2.32)	1.27 (0.93-1.72)	1.26 (0.92-1.71)
**South Central and East**	0.95 (0.60-1.51)	1.00 (0.63-1.59)	1.44 (0.62-3.38)	2.20 (0.88-5.54)	1.02 (0.68-1.52)	1.07 (0.71-1.61)
**South**	**1.80 (1.00-3.23)**	**1.79 (0.99-3.24)**	**3.06 (1.17-7.99)**	**5.43 (1.83-16.12)**	**2.00 (1.22-3.27)**	**2.03 (1.23-3.35)**
**Tumor sites**						
**Thyroid**	—	Ref	—	Ref	—	Ref
**Breast**	**—**	**0.43 (0.21-0.88)**	—	—	**—**	**0.37 (0.19-0.71)**
**Testicular**	—	—	**—**	**0.14 (0.02-0.84)**	**—**	**0.36 (0.16-0.81)**
**Ovarian**	—	0.45 (0.20-1.02)	—	—	**—**	**0.39 (0.18-0.86)**
**Cervical**	—	0.74 (0.36-1.53)	—	—	—	0.68 (0.34-1.34)
**Colorectal**	—	0.37 (0.13-1.08)	**—**	**0.01 (0.001-0.13)**	**—**	**0.18 (0.07-0.47)**
**Head and neck**	—	0.79 (0.29-2.16)	—	0.24 (0.03-2.29)	—	0.67 (0.28-1.64)
**Lymphoma**	**—**	**0.39 (0.16-0.93)**	—	0.26 (0.04-1.61)	**—**	**0.43 (0.20-0.91)**
**Leukemia**	—	0.70 (0.24-2.09)	**—**	**0.11 (0.01-1.00)**	—	0.54 (0.21-1.39)
**Other**	**—**	**0.40 (0.19-0.83)**	**—**	**0.18 (0.03-0.98)**	**—**	**0.36 (0.19-0.69)**
**Cancer stage**						
**Stage 0**	Ref	Ref	Ref	Ref	Ref	Ref
**Stage I**	1.27 (0.65-2.49)	1.24 (0.63-2.46)	2.51 (0.67-9.40)	2.30 (0.56-9.42)	1.50 (0.84-2.69)	1.42 (0.79-2.57)
**Stage II**	1.18 (0.61-2.27)	1.13 (0.58-2.21)	2.09 (0.53-8.14)	1.96 (0.46-8.35)	1.37 (0.77-2.43)	1.31 (0.73-2.34)
**Stage III**	1.03 (0.53-2.01)	1.02 (0.52-1.99)	2.35 (0.62-8.89)	2.95 (0.70-12.47)	1.28 (0.72-2.29)	1.27 (0.70-2.28)
**Stage IV**	1.25 (0.58-2.67)	1.19 (0.55-2.57)	2.94 (0.64-13.51)	2.15 (0.42-10.99)	1.48 (0.77-2.86)	1.33 (0.68-2.60)
**Unknown**	1.04 (0.56-1.94)	1.01 (0.53-1.91)	2.05 (0.58-7.25)	1.95 (0.52-7.34)	1.24 (0.72-2.13)	1.15 (0.66-2.01)
**Surgery**						
**No**	Ref	Ref	Ref	Ref	Ref	Ref
**Yes**	1.12 (0.75-1.67)	1.18 (0.72-1.94)	0.92 (0.48-1.78)	1.45 (0.63-3.34)	1.06 (0.76-1.48)	1.24 (0.82-1.87)
**Chemotherapy**						
**No**	Ref	Ref	Ref	Ref	Ref	Ref
**Yes**	1.02 (0.72-1.44)	1.39 (0.92-2.12)	1.24 (0.58-2.66)	2.05 (0.80-5.28)	1.05 (0.77-1.42)	**1.49 (1.04-2.15)**
**Radiotherapy**						
**No**	Ref	Ref	Ref	Ref	Ref	Ref
**Yes**	1.09 (0.81-1.46)	1.12 (0.82-1.54)	0.94 (0.50-1.75)	1.05 (0.52-2.11)	1.02 (0.79-1.32)	1.05 (0.79-1.38)
**Hormone therapy**						
**No**	Ref	Ref	Ref	Ref	Ref	Ref
**Yes**	1.38 (0.97-1.96)	1.45 (0.99-2.12)	4.93 (0.39-61.69)	1.29 (0.06-27.59)	1.38 (0.98-1.95)	**1.45 (1.00-2.11)**

Bolded: Statistically significant at *P* < .05.

a Anxiety is defined as No (did not endorse having symptoms on self-report checklist questionnaire) vs Yes (endorsed symptoms on checklist).

b Model 1 is adjusted for age at diagnosis, sex (when appropriate), time since end of treatment, education level, region, cancer stage, receipt of surgery, receipt of chemotherapy, receipt of radiotherapy, receipt of hormone therapy.

c Model 2 is adjusted for all Model 1 variables in addition to cancer type.

d Odds ratios (OR) and 95% CIs estimated from logistic regression modeling presence of anxiety symptoms (yes vs no). OR > 1 indicates a higher odds of reporting anxiety symptoms for that group in relation to the Reference group, OR < 1 indicates a lower odds.

### Associations of reported infertility with depression symptoms

Among Mexican AYA male and female cancer survivors combined, in univariable analysis those who endorsed infertility had a higher likelihood of experiencing depression symptoms (OR 1.66, 95% CI: 1.16-2.38) than those who did not endorse infertility. In multivariable analysis, adjusting for age at diagnosis, time since the end of treatment, education level, region, cancer stage, treatment received (Table 3, Total, Model 1) and furthermore cancer type (Table 3, Total, Model 2), an association persisted between reported infertility and depression (OR 1.52, 95% CI: 1.03-2.23; OR 1.52, 95% CI: 1.02-2.26, respectively). For women, those who reported infertility had a higher likelihood of depression than women who did not report infertility in both unadjusted (OR 1.70, 95% CI: 1.13-2.54) and adjusted (Table 3, Female, Model 1, OR 1.59, 95% CI: 1.03-2.44) analyses. This association was attenuated after additional adjustment for cancer type (Table 3, Female, Model 2, OR 1.53, 95% CI: 0.98-2.37). In comparison to women with thyroid cancer, those with breast (Table 3, Female, Model 2, OR 0.35, 95% CI: 0.16-0.73), ovarian (Table 3, Female, Model 2, OR 0.37, 95% CI: 0.16-0.87), and colorectal (Table 3, Female, Model 2, OR 0.26, 95% CI: 0.09-0.78) cancer as well as lymphoma (Table 3, Female, Model 2, OR 0.34, 95% CI: 0.14-0.83) were less likely to endorse depression symptoms. For men, reported infertility was not associated with experiencing depression symptoms in unadjusted (OR 1.21, 95% CI: 0.52-2.86) or adjusted (Table 3, Male, Model 1/2) analyses. Sensitivity analyses yielded similar results when age was restricted to those 18 and older (Tables S3 and S4).

**Table 3:** Predictors of depression^a^ symptoms in adolescents and young adults with cancer in Mexico, 2014-2018.

	Female		Male		Total	
	Model 1^b^	Model 2^c^	Model 1^b^	Model 2^c^	Model 1^b^	Model 2^c^
	OR (95% CI)^d^	OR (95% CI)^d^	OR (95% CI)^d^	OR (95% CI)^d^	OR (95% CI)^d^	OR (95% CI)^d^
**Infertility**						
**No**	Ref	Ref	Ref	Ref	Ref	Ref
**Yes**	**1.59 (1.03-2.44)**	1.53 (0.98-2.37)	1.49 (0.55-4.08)	1.72 (0.59-5.02)	**1.52 (1.03-2.23)**	**1.52 (1.02-2.26)**
**Sex**						
**Male**	—	—	—	—	Ref	Ref
**Female**	—	—	—	—	**1.96 (1.43-2.68)**	1.40 (0.93-2.11)
**Age at diagnosis (years)**						
**15-17**	Ref	Ref	Ref	Ref	Ref	Ref
**18-25**	0.66 (0.30-1.48)	0.70 (0.31-1.60)	2.61 (0.75-9.17)	**3.75 (1.00-14.03)**	1.05 (0.57-1.96)	1.16 (0.62-2.20)
**26-39**	0.54 (0.25-1.14)	0.61 (0.28-1.34)	2.28 (0.67-7.75)	3.15 (0.86-11.52)	0.88 (0.49-1.58)	0.98 (0.53-1.81)
**Time since end of treatment (years)**						
**<1**	Ref	Ref	Ref	Ref	Ref	Ref
**1-4**	**1.66 (1.06-2.62)**	**1.70 (1.07-2.71)**	0.80 (0.33-1.95)	0.93 (0.37-2.37)	1.46 (0.98-2.17)	**1.56 (1.04-2.34)**
**5-9**	1.40 (0.89-2.20)	1.46 (0.92-2.31)	1.11 (0.49-2.55)	1.21 (0.50-2.94)	1.34 (0.91-1.98)	1.38 (0.93-2.06)
**≥10**	**1.83 (1.16-2.89)**	**1.95 (1.22-3.11)**	1.23 (0.52-2.89)	1.28 (0.51-3.20)	**1.70 (1.14-2.53)**	**1.84 (1.23-2.76)**
**Education level**						
**College or higher**	Ref	Ref	Ref	Ref	Ref	Ref
**High school**	**2.04 (1.40-2.96)**	**1.98 (1.35-2.90)**	1.17 (0.61-2.24)	1.03 (0.52-2.04)	**1.84 (1.34-2.53)**	**1.78 (1.29-2.45)**
**Less than high school**	**2.00 (1.42-2.81)**	**2.00 (1.41-2.85)**	1.50 (0.72-3.11)	1.61 (0.73-3.53)	**1.84 (1.36-2.49)**	**1.89 (1.38-2.59)**
**Unknown**	0.51 (0.13-2.02)	0.42 (0.11-1.72)	0.32 (0.04-2.86)	0.29 (0.03-2.81)	0.47 (0.15-1.49)	0.40 (0.13-1.30)
**Region**						
**Northwest**	Ref	Ref	Ref	Ref	Ref	Ref
**Northeast**	0.97 (0.41-2.28)	1.11 (0.46-2.64)	0.28 (0.03-2.70)	0.30 (0.03-3.39)	0.79 (0.36-1.71)	0.87 (0.40-1.90)
**West and Lowlands**	1.10 (0.63-1.93)	1.11 (0.63-1.97)	1.08 (0.39-3.02)	1.11 (0.38-3.21)	1.06 (0.65-1.74)	1.07 (0.65-1.76)
**Mexico City**	1.26 (0.89-1.80)	1.27 (0.89-1.81)	0.90 (0.46-1.79)	0.93 (0.45-1.90)	1.17 (0.86-1.60)	1.18 (0.86-1.61)
**South Central and East**	1.29 (0.81-2.04)	1.34 (0.84-2.15)	1.24 (0.52-2.92)	1.70 (0.67-4.30)	1.17 (0.78-1.74)	1.25 (0.83-1.87)
**South**	1.58 (0.87-2.86)	1.56 (0.86-2.85)	2.14 (0.82-5.59)	**3.26 (1.14-9.30)**	1.55 (0.94-2.56)	1.59 (0.96-2.64)
**Tumor sites**						
**Thyroid**	—	Ref	—	Ref	—	Ref
**Breast**	**—**	**0.35 (0.16-0.73)**	—	—	**—**	**0.32 (0.16-0.63)**
**Testicular**	—	—	**—**	**0.15 (0.02-0.87)**	**—-**	**0.18 (0.08-0.42)**
**Ovarian**	**—**	**0.37 (0.16-0.87)**	—	—	**—**	**0.34 (0.15-0.76)**
**Cervical**	—	0.60 (0.28-1.28)	—	—	—	0.56 (0.27-1.14)
**Colorectal**	**—**	**0.26 (0.09-0.78)**	**—**	**0.05 (0.01-0.46)**	**—**	**0.16 (0.06-0.40)**
**Head and neck**	—	0.45 (0.16-1.26)	—	1.04 (0.12-9.08)	—	0.51 (0.20-1.26)
**Lymphoma**	**—**	**0.34 (0.14-0.83)**	—	0.41 (0.07-2.53)	**—**	**0.36 (0.17-0.78**)
**Leukemia**	—	0.39 (0.13-1.21)	—	0.14 (0.02-1.32)	**—**	**0.30 (0.12-0.79)**
**Other**	—	0.51 (0.24-1.08)	—	0.32 (0.06-1.81)	**—**	**0.44 (0.23-0.86)**
**Cancer stage**						
**Stage 0**	Ref	Ref	Ref	Ref	Ref	Ref
**Stage I**	1.14 (0.58-2.26)	1.08 (0.54-2.16)	1.01 (0.28-3.56)	0.99 (0.26-3.75)	1.15 (0.65-2.05)	1.08 (0.60-1.95)
**Stage II**	0.88 (0.45-1.71)	0.84 (0.43-1.65)	1.50 (0.42-5.32)	1.87 (0.49-7.12)	1.04 (0.59-1.85)	0.99 (0.55-1.76)
**Stage III**	1.02 (0.52-2.00)	1.00 (0.51-1.96)	1.27 (0.36-4.50)	1.48 (0.38-5.74)	1.15 (0.64-2.04)	1.10 (0.61-1.97)
**Stage IV**	1.03 (0.48-2.23)	0.96 (0.44-2.10)	3.64 (0.88-15.02)	3.01 (0.68-13.30)	1.45 (0.75-2.79)	1.26 (0.64-2.45)
**Unknown**	0.96 (0.51-1.81)	0.91 (0.48-1.74)	1.36 (0.42-4.41)	1.32 (0.39-4.47)	1.11 (0.65-1.91)	1.02 (0.59-1.76)
**Surgery**						
**No**	Ref	Ref	Ref	Ref	Ref	Ref
**Yes**	1.34 (0.89-2.00)	1.33 (0.81-2.18)	0.61 (0.32-1.17)	0.95 (0.43-2.10)	1.06 (0.76-1.48)	1.20 (0.80-1.81)
**Chemotherapy**						
**No**	Ref	Ref	Ref	Ref	Ref	Ref
**Yes**	0.78 (0.55-1.11)	1.13 (0.74-1.71)	0.81 (0.39-1.69)	1.39 (0.57-3.38)	0.76 (0.56-1.04)	1.18 (0.82-1.70)
**Radiotherapy**						
**No**	Ref	Ref	Ref	Ref	Ref	Ref
**Yes**	0.84 (0.62-1.13)	0.86 (0.63-1.18)	0.76 (0.41-1.40)	0.64 (0.33-1.27)	0.79 (0.61-1.02)	0.78 (0.59-1.03)
**Hormone therapy**						
**No**	Ref	Ref	Ref	Ref	Ref	Ref
**Yes**	1.24 (0.86-1.77)	1.35 (0.91-1.98)	5.91 (0.47-74.45)	3.50 (0.19-62.98)	1.31 (0.92-1.86)	1.39 (0.95-2.04)

Bolded: Statistically significant at *P* < .05.

a Depression is defined as No (did not endorse having symptoms on self-report checklist questionnaire) vs Yes (endorsed symptoms on checklist).

b Model 1 is adjusted for age at diagnosis, sex (when appropriate), time since end of treatment, education level, region, cancer stage, receipt of surgery, receipt of chemotherapy, receipt of radiotherapy, receipt of hormone therapy.

c Model 2 is adjusted for all Model 1 variables in addition to cancer type.

d Odds ratios (OR) and 95% CIs estimated from logistic regression modeling presence of depression symptoms (yes vs no). OR > 1 indicates a higher odds of reporting anxiety symptoms for that group in relation to the Reference group, OR < 1 indicates a lower odds.

## Discussion

Our primary aim was to determine the association between infertility, anxiety, and depression among female and male AYA cancer survivors in Mexico. Infertility was found to be associated with depression, but not anxiety, among AYA Mexican cancer survivors. Mexican AYA cancer survivors experiencing infertility may need additional support to address unmet supportive care needs. These findings build upon previous research examining reproductive issues and psychological distress among breast cancer survivors and extend this work to female and male AYA cancer survivors with both reproductive and nonreproductive-related cancers in low-resource settings such as Mexico.

Our findings highlight the burden of anxiety and depression among AYA cancer survivors in Mexico. In our study, 42% and 46% of all AYA cancer survivors report anxiety and depression symptomology, respectively. This prevalence is remarkably higher than the latest estimates from the 2016-2017 National Survey of Drug, Alcohol, and Tobacco Use (Encuesta Nacional de Consumo de Drogas, Alcohol y Tabaco, ENCODAT) which determined that 4% and 6% of the general population (ages 12-65) in Mexico experienced anxiety and depression, respectively.^21^ The burden of anxiety and depression amongst this sample of Mexican AYA cancer survivors is also substantially elevated in comparison to other populations of AYA cancer survivors, with a recent meta-analysis demonstrating a pooled prevalence of 29% and 24% of anxiety and depression.^22^

Endorsing infertility was associated with depression symptoms among Mexican AYAs, likely driven largely by women. Our findings are consistent with past studies by Gorman et al.^23^^,^^24^ which identified a relationship between reproductive concerns and depressive symptoms after controlling for sociodemographic and clinical factors as well as social support among not only young breast cancer survivors but female cancer survivors in general. Barriers to adequate provision of cancer-related information (ie, treatment, symptoms, self-management advice and support, health care system navigation, and care coordination) may partly explain the relationship between reproductive concerns and depression.^25^^,^^26^ Among cancer survivors, prospective studies have shown that a lack of information, barriers to information provision, or information of low quality or clarity is associated with increased anxiety, depression, and decreased health-related quality of life.^26^ In our study, lack of information on infertility risk, fertility preservation options, procedures, and how to navigate attaining services can be pivotal. Additionally, considering the cultural values of “familismo” (ie, the strong identification of individuals with family members and alongside loyalty, interdependence, solidarity, and collective support)^27^^,^^28^ and “marianismo” (ie, emphasis of traditional female gender roles related to family and home centeredness, including chastity, passivity, and self-sacrifice)^29^^,^^30^ within Mexican culture, fertility-related issues may have an even more profound impact on quality of life, due to stigma, familial pressure, and complicated marital dynamics due to gender roles.^31^^,^^32^

The association between reported infertility and anxiety and depression symptoms in female AYAs in Mexico was attenuated when adjusting for cancer type. This is likely due to a lack of statistical power when adjusting for many cancer types, more than ten, in our study. In comparison to female AYAs with thyroid cancer, women with breast cancer and lymphoma were less likely to experience both anxiety and depression symptoms, and additionally, those with ovarian and colorectal cancer also had a lower likelihood of endorsing anxiety symptoms. These findings may reflect sociodemographic and clinical differences in patient characteristics, such as an older age at diagnosis, longer time since treatment completion, and lower fear of cancer recurrence among this sample of female AYA cancer survivors with breast cancer in Mexico. Given the current focus on patients with breast or gynecological cancers, few studies have assessed how the relationship between infertility and psychological distress varies by cancer site and findings are conflicting. While some studies have observed no differences by cancer type^33^ others found that having a cancer diagnosis involving or close to the reproductive organs was associated with heightened anxiety.^9^^,^^34^ Meanwhile, women with nonreproductive cancer types may have lower acceptance of cancer and also reported a higher importance of parenthood.^10^ Additionally, studies have demonstrated that the likelihood of receiving fertility-related information, both about the potential impact of treatment on fertility and fertility preservation options, varies across cancer type.^13^^,^^35^ Women with breast, cervical, and ovarian cancer are more likely to be informed.^35^ Considering Mexico’s healthcare infrastructure, differences in cancer awareness by tumor type and resource allocation may impact care priorities. Breast cancer may often times be treated within multidisciplinary oncology units with integrated psychological support, while thyroid cancer may be managed with less intensive follow-up, potentially limiting access to psychosocial care. Together, these factors could influence cancer preparedness related to issues regarding fertility-related concerns.

The association between infertility and anxiety and depression found among Mexican male AYA cancer survivors was not as strong as that observed among women. In general, case-control studies have demonstrated that men with diagnosed infertility were found to have significantly higher levels of depression and anxiety than those without infertility.^36^ These studies have been primarily focused on males throughout the United States, Asia, and Europe but not Latin America. Furthermore, limited studies have assessed infertility and psychological distress among the male cancer survivor population, specifically. Stigma related to mental health as well as gendered cultural norms pertaining to masculinity and the role of “machismo” may play a role in Mexico.^37^^,^^38^ In a study examining help-seeking behaviors for mental-health-related concerns in Mexico, over a quarter of participants indicated self-stigma.^38^ Furthermore, research has demonstrated that men are often more likely to endorse feelings of irritability, anger, and frustration as manifestations of psychological anxiety and depression, possibly due to socialization and norms around social expression.^39–41^ These factors can lead to reporting biases and underestimation of infertility, anxiety, and depression symptoms, which may partially account for the findings of our study, particularly amongst men. For example, in our study, 13% of female Mexican AYAs reported infertility, which is comparable to similar studies examining self-reported infertility amongst adult survivors of childhood cancer in the US.^42^ However, only 9% of male AYAs endorsed infertility, which is much lower than the 46% cited by previous studies of male childhood cancer survivors.^43^ Limited data exist on infertility rates in Mexico’s general population. One study of reproductive-aged women across 12 states in Mexico enrolled in the Mexican Teachers’ Cohort found infertility affected 17% of women, higher than the 13% reported in the US general population and global estimate for low- and middle-income countries.^44–46^ To our knowledge, no data exist on male infertility in Mexico. Variability in how infertility is defined and measured in the literature makes comparisons challenging.

Our findings must be interpreted within the context of the sociopolitical, economic, and cultural climate of Mexico. The mental health crisis in Mexico is multifaceted given the very limited resources, with only 2% of the health budget being allocated to mental health services, of which 80% are solely dispersed to psychiatric institutions,^47^ with little focus on prevention, diagnosis, and management of psychological conditions.^48^ As such, the economic and cultural barriers to accessing and receiving mental health care include but are not limited to the availability of services, beliefs surrounding mental health, religiosity, and lack of faith in the health system.^38^ Given the rise of psychosocial needs during the COVID-19 pandemic,^49^ an integrated system incorporating multidisciplinary oncology teams that include mental health providers will become even more important moving forward in order to reduce stigma and provide high-quality cancer care.^50^ Additionally, the oncofertility infrastructure in Mexico remains underdeveloped.^51^ Currently, Mexico does not have a centralized cancer registry or a national oncofertility registry.^52^ Although Mexico does have the technological medical advances to offer various fertility preservation options, there is a lack of institutional and research funds, a lack of health insurance coverage for fertility services, and fertility treatments are provided exclusively in private centers.^52^ Geographic disparities exist in oncology care across Mexico, with Central (eg, Mexico City) and Northern regions having the highest concentration of healthcare resources, including private hospitals, oncology centers, and specialists.^53–55^ In contrast, Southern and rural regions have fewer radiotherapy centers, equipment, and oncologists, leading to unequal access to quality cancer care.^54^^,^^55^ These disparities likely extend to fertility preservation, with patients in less-resourced areas facing additional barriers to accessing oncofertility counseling and treatment options. Socioeconomic inequities, such as lower health insurance coverage and higher poverty rates in rural populations, may further limit access to these services.^53^^,^^55^ Oncofertility knowledge is also low amongst oncology providers in Mexico, with 50% of providers reporting not being able to name a fertility preservation method and only 10% reporting self-perceived confidence in providing fertility preservation counseling.^17^

**Figure 1:**
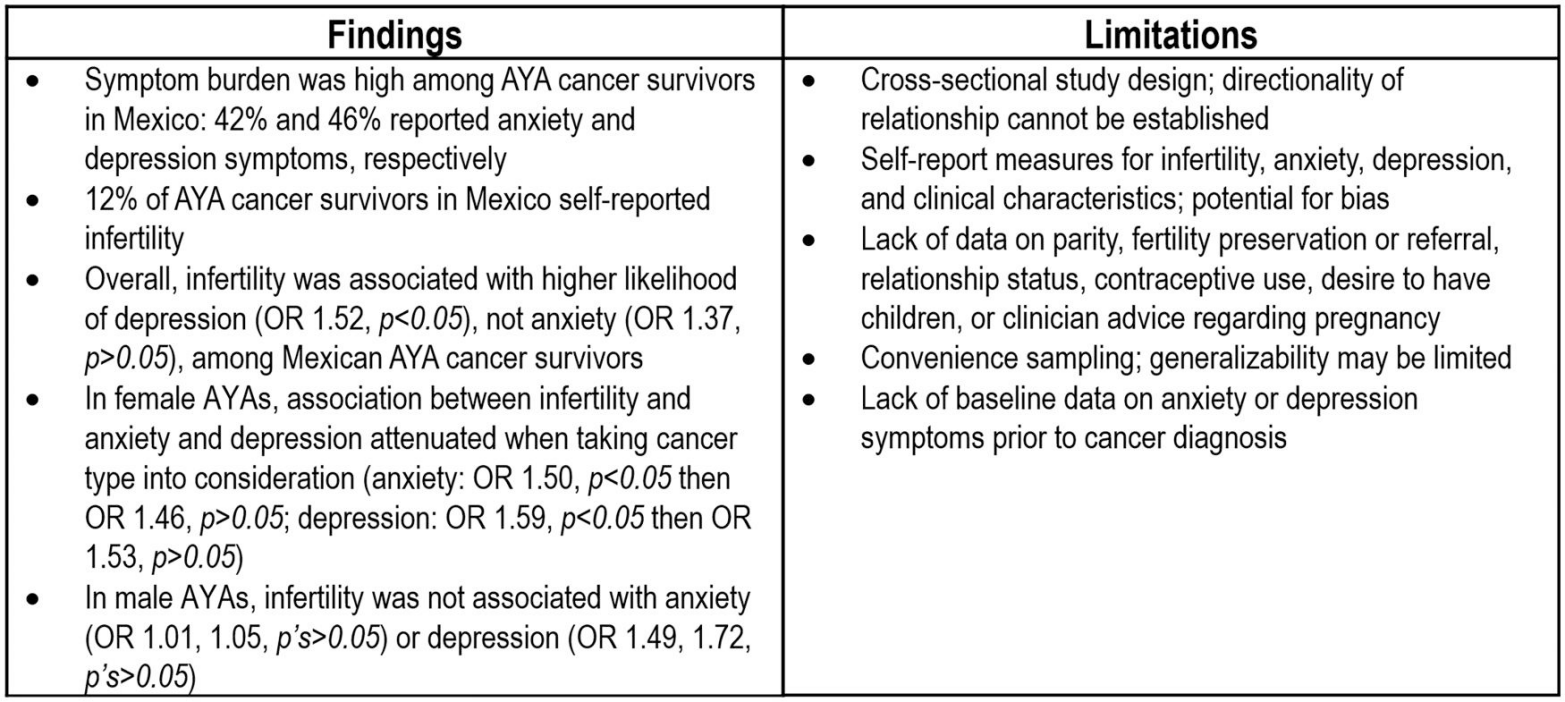
Summary of key findings and limitations.

Key findings and limitations are summarized in Figure 1. To our knowledge, this is the first study to focus on infertility and anxiety and depression among male and female AYAs across Mexico. This survey included data from a large diverse nationwide sample of AYAs from all regions in Mexico with varying cancer types. The majority of research on fertility concerns among AYA cancer survivors has focused on non-Hispanic/Latino White, and to a smaller degree, Hispanic/Latino populations in high-income countries. Cultural values of “familismo” and “marianismo” may heighten the psychological burden of infertility among Hispanic/Latino populations. For example, qualitative studies have shown that Hispanic/Latina women often perceive cultural and social pressure to conceive, with infertility affecting their sense of belonging and identity.^32^ These cultural influences may not be as prominent in non-Hispanic/Latino populations but could have parallels in other cultures where family expectations and reproductive norms strongly shape personal well-being. The generalizability of these findings should be interpreted with cultural and healthcare system differences in mind. Furthermore, there was a wealth of not only cancer-specific clinical and treatment data but also sociodemographic and psychosocial factors which are often not present in cancer registry data.

When interpreting the findings of this study, some limitations need to be considered. Given the cross-sectional nature of this data, a temporal relationship between infertility and anxiety and/or depression cannot be established. Additionally, the use of self-report measures for infertility, anxiety, depression, and all clinical characteristics may introduce bias. Self-reported infertility, lacking clinical evaluation, may introduce bias as some patients may not meet clinical criteria but instead choose to delay or forgo pregnancy due to medical advice on cancer treatment risks or prognosis concerns. Due to the lack of data on whether cancer survivors had children prior to their diagnosis, we cannot distinguish between primary infertility (no prior pregnancy) and secondary infertility (inability to conceive or carry a baby to term after a previous birth), an important distinction given the limited research on secondary infertility. We also did not have information regarding whether patients underwent fertility preservation or were referred to services at INCAN or other centers/hospitals in Mexico. Future studies should replicate findings using clinically confirmed infertility diagnoses while incorporating information regarding relationship status, birth history, contraceptive use, desire to have a child, and medical advice from healthcare professionals. They should also cross-validate self-report tumor-related characteristics with electronic medical records and use validated measures of anxiety, depression, quality of life, and other psychosocial factors. As such, recall bias could impact the findings of this study due to the self-report nature. However, although this information was measured post-treatment completion, research has indicated that fertility-related psychological distress may be present for up to 20 years post-diagnosis.^9^^,^^56^ This survey was also conducted via convenience sampling, and therefore it is possible that those cancer survivors who volunteered to participate may not be representative of all cancer survivors in Mexico. Like many cancer survivorship studies, we lack baseline data on anxiety or depression prior to diagnosis, limiting our ability to distinguish cancer-related distress from pre-existing anxiety or depression, highlighting the need for future population-based studies across the lifespan.

This current study sheds light on the relationship between self-reported infertility and anxiety/depression symptomatology among AYA cancer survivors in Mexico. These findings underscore the pressing need to address the ever-changing healthcare landscape within the country, which encompasses a lack of a robust mental health and oncofertility infrastructure. As Mexico grapples with increasing rates of cancer diagnoses among its younger population, it becomes imperative to prioritize comprehensive cancer care that encompasses both physical and psychological well-being and caters to the unique needs of AYAs. The lack of resources and support for fertility preservation among cancer patients exacerbates the already challenging journey they face. Integrating mental health support and fertility preservation services into existing oncological care frameworks can help mitigate the adverse psychosocial effects of cancer treatment and improve the overall quality of life for this vulnerable population in Mexico. Multidisciplinary care models, including reproductive endocrinologists, psychologists, and social workers, can improve patient outcomes, particularly as AYAs transition out of oncology care with limited follow-up support for fertility and mental health concerns. Long-term survivorship guidelines are needed to improve care coordination and referrals for AYA cancer survivors. Further investigation into reproductive concerns, psychological distress, and financial toxicity is needed among Mexican AYAs.

## Supplementary Material

oyag062_Supplementary_Data

## Data Availability

The authors confirm that, for approved reasons, some access restrictions apply to the data underlying the findings. Deidentified data from this study are not available in a public archive. Deidentified data from this study will be made available (as allowable according to institutional standards) on request from the corresponding author and the Instituto Nacional de Cancerología in Mexico.
