# Supplementary material for: Infertility, anxiety, and depression among adolescents and young adults with cancer: the Mexico Cancer Survivorship Registry
Source: Oncologist. 2026 Mar 9;31(4):oyag062. doi: 10.1093/oncolo/oyag062 (PMC13006059; doi:10.1093/oncolo/oyag062)
Supplement: oyag062_Supplementary_Data [file oyag062_supplementary_data.zip › Supplementary_Table_1.docx]

**Supplementary Table 1:** Registro de Supervivientes de Cancer Survey Questionnaire in Spanish.

| BLOQUE 1  La información obtenida del Registro de Supervivientes de Cáncer servirá para desarrollar programas y ofrecer servicios que apoyen más efectivamente a los supervivientes. | |
| --- | --- |
| Estado: | Ciudad: |
| Delegación o municipio: |  |
| Sexo: | F |
|  | M |
| Fecha de nacimiento: | dd/mm/aaaa |
| ¿Qué tipo de cáncer tuvo? | Cervicouterino |
|  | Colorrectal |
|  | Endometrio |
|  | Esófago |
|  | Hígado |
|  | Laringe e hipofaringe |
|  | Leucemia |
|  | Linfoma |
|  | Mama |
|  | Mieloma |
|  | Primario desconocido |
|  | Orofaringe y cavidad oral |
|  | Ovario |
|  | Páncreas |
|  | Piel |
|  | Próstata |
|  | Pulmón |
|  | Riñón |
|  | Sarcoma de hueso (osteosarcoma) |
|  | Sarcoma de tejidos blandos |
|  | Testículo |
|  | Tiroides |
|  | Vagina |
|  | Vejiga |
|  | Vesícula biliar |
|  | Otro: |
| ¿En qué fecha se lo diagnosticaron? | dd/mm/aaaa |
| ¿En qué fecha inició con el tratamiento? | dd/mm/aaaa |
| ¿En qué fecha terminó su tratamiento?*  *Cuando le dijeron que estaba libre de la enfermedad (ya no tenía cáncer) | dd/mm/aaaa |

| BLOQUE 2  Este Registro de Supervivientes de Cáncer es un mecanismo por el cual los profesionales de la salud pueden conocer las experiencias de los pacientes durante y después del tratamiento. | |
| --- | --- |
| ¿En qué etapa clínica le diagnosticaron el cáncer? | 0 *(in situ*) |
|  | I |
|  | II |
|  | III |
|  | IV |
|  | No lo recuerdo |
| ¿En qué hospital se trató? |  |
| ¿Qué tipo de tratamiento recibió? Marque todos los tratamientos que le administraron (puede marcar más de una opción) | Cirugía |
|  | Quimioterapia |
|  | Radioterapia interna (Braquiterapia) |
|  | Radioterapia |
|  | Hormonoterapia |
|  | Anticuerpos monoclonales (Herceptin, Rituxan, Cetuximab, Panitumumab) |
|  | Yodo radioactivo |
|  | Trasplante de médula ósea |
|  | Otros: |
| ¿Acude a sus citas de seguimiento? | Sí |
|  | No |
| ¿Se realiza los estudios de seguimiento que le indica su médico? | Sí |
|  | No |
| ¿Actualmente lleva su seguimiento en la misma Institución donde lo trataron? | Sí |
|  | No |

| BLOQUE 3  El principal objetivo del Registro de Supervivientes de Cáncer es la colección y análisis de información relacionada con las necesidades físicas, emocionales y sociales de los pacientes después de la enfermedad. | | |
| --- | --- | --- |
| ¿Cómo califica su estado de salud en general? | Muy bueno | |
|  | Bueno | |
|  | Regular | |
|  | Malo | |
| ¿Ha presentado alguno de los siguientes efectos secundarios? Puede seleccionar más de una opción. | Aumento de peso | |
|  | Cambios en la piel | |
|  | Cardiopatías (problemas del corazón) | |
|  | Trastornos dentales (de los dientes) | |
|  | Dificultad respiratoria | |
|  | Alteraciones digestivas | |
|  | Disfunción sexual | |
|  | Dolor de huesos | |
|  | Fatiga | |
|  | Hipotiroidismo (trastorno de la glándula tiroides) | |
|  | Incontinencia (pérdida del control de la orina) | |
|  | Infertilidad (incapacidad para lograr un embarazo) | |
|  | Linfedema (inflamación de brazos o piernas debido a acumulación de líquido) | |
|  | Neuropatía (dolor, adormecimiento, hormigueos, hinchazón y debilidad muscular en varias partes del cuerpo) | |
|  | Osteoporosis (fragilidad de los huesos) | |
|  | Pérdida de memoria | |
|  | Problemas hormonales (trastornos que provocan pérdida o aumento de peso, cansancio, bochornos, entre otros) | |
|  | Pérdida de peso (reducción de masa corporal) | |
|  | Problemas de la visión (vista) | |
|  | Otros: | |
| ¿Ha tenido alguna de las siguientes alteraciones psicosociales? Puede marcar más de una opción. | Depresión | |
|  | Temor por una recaída (regreso del cáncer) | |
|  | Ansiedad | |
|  | Preocupación | |
|  | Problemas con las relaciones familiares | |
|  | Problemas económicos | |
|  | Estrés (tensión nerviosa) de vivir con un antecedente de cáncer | |
|  | Inseguridad (incertidumbre) | |
|  | Otros: | |
| ¿Ha sufrido alguno de los siguientes efectos en su sexualidad? Puede marcar más de una opción. | Disminución del deseo sexual (falta de ganas de tener relaciones sexuales) | |
|  | Disfunción eréctil o eyaculación precoz (impotencia) | |
|  | Miedo a no hacer un buen papel al momento del acto sexual (no funcionar) | |
|  | Dolor durante las relaciones sexuales | |
|  | Rechazo de su pareja | |
|  | Otro: | |
| ¿Actualmente procura tener una alimentación balanceada que incluya frutas, verduras, cereales y carne? | Sí | |
|  | No | |
| ¿Mantiene un peso adecuado de acuerdo con su edad y complexión física? | Sí | |
|  | No | |
| ¿Realiza alguna actividad física? | Sí | ¿Cuál? |
|  | No | |
| ¿Fuma actualmente? | Sí | |
|  | No | |
| ¿Ingiere bebidas alcohólicas? | Sí | |
|  | No | |

| BLOQUE 4  Incluir en el Registro de Supervivientes de Cáncer información acerca de aspectos sociales permitirá mejorar la calidad de vida de las personas que sobreviven a esta enfermedad. | | |
| --- | --- | --- |
| ¿Durante o después de su tratamiento recibió apoyo de alguna asociación de beneficencia o institución de la sociedad civil? | Sí | ¿Cuál? |
|  | No | |
| ¿Actualmente participa con alguna organización que apoye a pacientes con cáncer o a otras causas como la ecología, violencia intrafamiliar, educación, etc.? | Sí | ¿Cuál? |
|  | No | |
| ¿Ha tenido dificultades para reincorporarse a sus actividades dentro de la familia o en el trabajo? | Sí | ¿Cuál? |
|  | No | |
| ¿Ha recibido algún tipo de apoyo psicológico? | Sí | Apoyo individual |
|  |  | Apoyo de grupo |
|  | No | |
| ¿Se ha sentido discriminado (no tratado igual que otras personas)? | Sí | |
|  | No | |

| BLOQUE 5  El Registro de Supervivientes de Cáncer también contribuirá a desarrollar programas educativos, actividades de prevención y políticas públicas relacionadas con los supervivientes de cáncer. | |
| --- | --- |
| ¿Hasta qué año estudió? | Ninguno |
|  | Primaria |
|  | Secundaria |
|  | Preparatoria |
|  | Licenciatura |
|  | Maestría |
|  | Otro: |
| ¿Cuál es su ocupación actual? |  |
| ¿Cómo se enteró de este Registro? | Periódicos |
|  | Televisión o radio |
|  | Redes Sociales |
|  | Sitio Web |
|  | Recomendación de un amigo |
|  | Recomendación de alguna institución |
|  | Algún otro medio |
