# Supplementary material for: Infertility, anxiety, and depression among adolescents and young adults with cancer: the Mexico Cancer Survivorship Registry
Source: Oncologist. 2026 Mar 9;31(4):oyag062. doi: 10.1093/oncolo/oyag062 (PMC13006059; doi:10.1093/oncolo/oyag062)
Supplement: oyag062_Supplementary_Data [file oyag062_supplementary_data.zip › Supplementary_Table_2.docx]

**Supplementary Table 2:** Registro de Supervivientes de Cancer Survey Questionnaire in English Translation.

| BLOCK 1  The information obtained from the Cancer Survivor Registry will be used to develop programs and provide services that more effectively support survivors. | |
| --- | --- |
| State: | City: |
| Delegation or Municipality: |  |
| Sex: | F |
|  | M |
| Date of Birth: | dd/mm/yyyy |
| What type of cancer did you have? | Cervix |
|  | Colorectal |
|  | Endometrium |
|  | Esophagus |
|  | Liver |
|  | Larynx and hypopharynx |
|  | Leukemia |
|  | Lymphoma |
|  | Breast |
|  | Myeloma |
|  | Unknown Primary |
|  | Oropharynx and Oral Cavity |
|  | Ovary |
|  | Pancreas |
|  | Skin |
|  | Prostate |
|  | Lung |
|  | Kidney |
|  | Bone Sarcoma (Osteosarcoma) |
|  | Soft Tissue Sarcoma |
|  | Testicular |
|  | Thyroid |
|  | Vaginal |
|  | Bladder |
|  | Gallbladder |
|  | Other: |
| On what date were you diagnosed? | dd/mm/yyyy |
| On what date did you start treatment? | dd/mm/yyyy |
| On what date did you finish your treatment?*  *When you were told you were free of the disease (no longer had cancer) | dd/mm/yyyy |

| BLOCK 2  This Cancer Survivor Registry is a mechanism by which health professionals can learn about patients’ experiences during and after treatment. | |
| --- | --- |
| At what clinical stage was your cancer diagnosed? | 0 *(in situ*) |
|  | I |
|  | II |
|  | III |
|  | IV |
|  | I do not remember |
| At which hospital did you receive treatment? |  |
| What type of treatment did you receive? Check all the treatments you received (you can check more than one option) | Surgery |
|  | Chemotherapy |
|  | Internal Radiotherapy (Brachytherapy) |
|  | Radiotherapy |
|  | Hormone Therapy |
|  | Monoclonal Antibodies (Herceptin, Rituxan, Cetuximab, Panitumumab) |
|  | Radioactive Iodine |
|  | Bone Marrow Transplant |
|  | Others: |
| Do you attend your follow-up visits? | Yes |
|  | No |
| Do you undergo the follow-up tests recommended by your doctor? | Yes |
|  | No |
| Are you currently receiving follow-up care at the same institution where you were treated? | Yes |
|  | No |

| BLOCK 3  The main objective of the Cancer Survivor Registry is to collect and analyze information related to the physical, emotional, and social needs of patients after the completion of treatment. | | |
| --- | --- | --- |
| How do you rate your overall health? | Excellent | |
|  | Good | |
|  | Fair | |
|  | Poor | |
| Have you experienced any of the following side effects? You may select more than one option. | Weight gain | |
|  | Skin changes | |
|  | Cardiopathies (heart problems) | |
|  | Dental (tooth) disorders | |
|  | Respiratory difficulties | |
|  | Digestive disorders | |
|  | Sexual Dysfunction | |
|  | Bone Pain | |
|  | Fatigue | |
|  | Hypothyroidism (disorder of the thyroid gland) | |
|  | Incontinence (loss of urine control) | |
|  | Infertility (inability to achieve pregnancy) | |
|  | Lymphedema (inflammation of arms or legs due to fluid accumulation) | |
|  | Neuropathy (pain, numbness, tingling, inflammation and muscle weakness in several parts of the body) | |
|  | Osteoporosis (bone fragility) | |
|  | Memory loss | |
|  | Hormonal problems (disorders that cause weight loss or gain, fatigue, hot flashes, etc.) | |
|  | Weight loss (reduction of body mass) | |
|  | Vision (eyesight) problems | |
|  | Others: | |
| Have you experienced any of the following psychosocial problems? You can choose more than one option. | Depression | |
|  | Fear of relapse (return of cancer) | |
|  | Anxiety | |
|  | Concern | |
|  | Problems with family relationships | |
|  | Financial problems | |
|  | Stress (nervous tension) of living with a history of cancer | |
|  | Insecurity (uncertainty) | |
|  | Other: | |
| Have you experienced any of the following effects on your sexuality? You can check more than one option. | Decreased sex drive (lack of desire to have sex) | |
|  | Erectile dysfunction or premature ejaculation (impotence) | |
|  | Fear of not performing well during sexual activity (not functioning) | |
|  | Pain during sexual intercourse | |
|  | Rejection by your partner | |
|  | Other: | |
| Do you currently try to maintain a balanced diet that includes fruits, vegetables, grains, and meat? | Yes | |
|  | No | |
| Do you maintain a healthy weight according to your age and body frame? | Yes | |
|  | No | |
| Do you engage in any physical activity? | Yes | Which one? |
|  | No | |
| Do you currently smoke? | Yes | |
|  | No | |
| Do you drink alcoholic beverages? | Yes | |
|  | No | |

| BLOCK 4  Including information about social aspects in the Cancer Survivors Registry will improve the quality of life of cancer survivors. | | |
| --- | --- | --- |
| During or after your treatment, did you receive support from a charitable organization or a civil society institution? | Yes | Which one? |
|  | No | |
| Are you currently involved with any organization that supports cancer patients or other causes such as environmental protection, domestic violence, education, etc.? | Yes | Which one? |
|  | No | |
| Have you had difficulty reintegrating into your family activities or work? | Yes | Which one? |
|  | No | |
| Have you received any type of psychological support? | Yes | Individual support |
|  |  | Group support |
|  | No | |
| Have you felt discriminated against (not treated the same as other people)? | Yes | |
|  | No | |

| BLOCK 5  The Cancer Survivor Registry will also contribute to the development of educational programs, prevention activities, and public policy related to cancer survivors. | |
| --- | --- |
| What is the highest level of education you completed? | None |
|  | Elementary School |
|  | Middle School |
|  | High School |
|  | Bachelor’s Degree |
|  | Master’s Degree |
|  | Other: |
| What is your current occupation? |  |
| How did you learn about this Registry? | Newspapers |
|  | Television or radio |
|  | Social media |
|  | Website |
|  | Recommendation from a friend |
|  | Recommendation from an institution |
|  | Some other means |
