# Supplementary material for: Infertility, anxiety, and depression among adolescents and young adults with cancer: the Mexico Cancer Survivorship Registry
Source: Oncologist. 2026 Mar 9;31(4):oyag062. doi: 10.1093/oncolo/oyag062 (PMC13006059; doi:10.1093/oncolo/oyag062)
Supplement: oyag062_Supplementary_Data [file oyag062_supplementary_data.zip › Supplementary_Table_3.docx]

| **Supplementary Table 3. Predictors of Anxiety**^a^ **Symptoms in Adolescents and Young Adults with Cancer in Mexico, 2014-2018.** | | | | | | |
| --- | --- | --- | --- | --- | --- | --- |
|  | **Female** | | **Male** | | **Total** | |
|  | **Model 1**^b^  OR (95% CI)^d^ | **Model 2**^c^  OR (95% CI)^d^ | **Model 1**^b^  OR (95% CI)^d^ | **Model 2^c^**  OR (95% CI)^d^ | **Model 1**^b^  OR (95% CI)^d^ | **Model 2**^c^  OR (95% CI)^d^ |
|  |  |  |  |  |  |  |
| **Infertility** |  |  |  |  |  |  |
| No | REF | REF | REF | REF | REF | REF |
| Yes | 1.40 (0.92-2.13) | 1.37 (0.89-2.11) | 0.94 (0.33-2.69) | 0.97 (0.32-2.94) | 1.31 (0.90-1.92) | 1.28 (0.87-1.91) |
| **Sex** |  |  |  |  |  |  |
| Male | -- | -- | -- | -- | REF | REF |
| Female | -- | -- | -- | -- | **1.66 (1.20-2.30)** | 1.38 (0.90-2.13) |
| **Age at Diagnosis (Years)** |  |  |  |  |  |  |
| 18-25 | REF | REF | REF | REF | REF | REF |
| 26-39 | 0.85 (0.57-1.25) | 0.90 (0.60-1.36) | 1.53 (0.83-2.83) | 1.76 (0.90-3.45) | 1.05 (0.76-1.45) | 1.11 (0.79-1.56) |
| **Time Since End of Treatment (Years)** |  |  |  |  |  |  |
| <1 | REF | REF | REF | REF | REF | REF |
| 1-4 | 1.16 (0.74-1.81) | 1.17 (0.74-1.85) | 0.85 (0.35-2.09) | 0.96 (0.37-2.54) | 1.14 (0.77-1.69) | 1.19 (0.80-1.78) |
| 5-9 | 1.07 (0.69-1.68) | 1.08 (0.69-1.71) | 0.90 (0.38-2.13) | 1.02 (0.39-2.65) | 1.09 (0.74-1.61) | 1.10 (0.74-1.63) |
| ≥10 | 0.98 (0.62-1.56) | 1.02 (0.64-1.62) | 0.80 (0.32-1.99) | 0.77 (0.29-2.06) | 0.98 (0.66-1.46) | 1.02 (0.68-1.54) |
| **Education Level** |  |  |  |  |  |  |
| College or Higher | REF | REF | REF | REF | REF | REF |
| High School | **1.58 (1.08-2.31)** | **1.55 (1.05-2.29)** | 0.67 (0.33-1.34) | 0.61 (0.29-1.29) | 1.30 (0.94-1.80) | 1.27 (0.91-1.77) |
| Less than High School | **1.46 (1.03-2.06)** | **1.43 (1.00-2.04)** | 0.72 (0.32-1.62) | 0.94 (0.39-2.28) | 1.26 (0.93-1.72) | 1.30 (0.95-1.80) |
| Unknown | 0.36 (0.07-1.81) | 0.31 (0.06-1.62) | 0.35 (0.04-3.14) | 0.46 (0.05-4.47) | 0.33 (0.09-1.20) | 0.31 (0.08-1.15) |
| **Region** |  |  |  |  |  |  |
| Northeast | REF | REF | REF | REF | REF | REF |
| Northwest | 1.65 (0.70-3.86) | 1.91 (0.81-4.52) | -- | -- | 1.19 (0.55-2.57) | 1.39 (0.63-3.04) |
| West and Lowlands | **1.92 (1.09-3.38)** | **1.94 (1.10-3.43)** | 1.04 (0.34-3.15) | 1.00 (0.30-3.26) | **1.68 (1.02-2.74)** | **1.69 (1.03-2.79)** |
| Mexico City | 1.42 (0.99-2.03) | 1.42 (0.99-2.04) | 1.10 (0.54-2.25) | 1.12 (0.52-2.38) | **1.36 (0.99-1.87)** | 1.36 (0.99-1.87) |
| South Central and East | 1.08 (0.68-1.73) | 1.13 (0.70-1.81) | 1.88 (0.77-4.60) | **2.97 (1.11-7.93)** | 1.18 (0.78-1.79) | 1.24 (0.82-1.89) |
| South | **1.88 (1.04-3.40)** | **1.89 (1.04-3.45)** | **3.33 (1.23-9.04)** | **6.18 (1.98-19.32)** | **2.10 (1.27-3.48)** | **2.15 (1.28-3.59)** |
| **Tumor Sites** |  |  |  |  |  |  |
| Thyroid | -- | REF | -- | REF | -- | REF |
| Breast | -- | **0.41 (0.19-0.86)** | -- | -- | -- | **0.36 (0.18-0.72)** |
| Testicular | -- | -- | -- | **0.15 (0.03-0.90)** | -- | **0.33 (0.14-0.77)** |
| Ovarian | -- | **0.40 (0.17-0.96)** | -- | -- | -- | **0.36 (0.16-0.82)** |
| Cervical | -- | 0.70 (0.33-1.48) | -- | -- | -- | 0.65 (0.32-1.32) |
| Colorectal | -- | **0.32 (0.11-0.99)** | -- | **0.01 (0.00-0.13)** | -- | **0.16 (0.06-0.42)** |
| Head & Neck | -- | 0.67 (0.23-1.92) | -- | 0.46 (0.05-4.72) | -- | 0.66 (0.26-1.70) |
| Lymphoma | -- | **0.36 (0.15-0.89)** | -- | 0.24 (0.04-1.51) | -- | **0.40 (0.18-0.86)** |
| Leukemia | -- | 0.87 (0.27-2.80) | -- | 0.11 (0.01-1.08) | -- | 0.62 (0.23-1.67) |
| Other | -- | **0.41 (0.19-0.89)** | -- | **0.18 (0.03-1.01)** | -- | **0.37 (0.19-0.74)** |
| **Cancer Stage** |  |  |  |  |  |  |
| Stage 0 | REF | REF | REF | REF | REF | REF |
| Stage I | 1.23 (0.63-2.42) | 1.22 (0.61-2.43) | 1.85 (0.47-7.24) | 1.72 (0.40-7.37) | 1.39 (0.77-2.51) | 1.33 (0.73-2.44) |
| Stage II | 1.08 (0.56-2.11) | 1.05 (0.53-2.06) | 1.93 (0.48-7.76) | 1.92 (0.44-8.45) | 1.24 (0.69-2.23) | 1.18 (0.65-2.14) |
| Stage III | 1.00 (0.51-1.96) | 1.00 (0.51-1.97) | 1.73 (0.44-6.86) | 2.39 (0.54-10.62) | 1.17 (0.65-2.11) | 1.18 (0.65-2.14) |
| Stage IV | 1.22 (0.56-2.63) | 1.15 (0.52-2.53) | 2.69 (0.57-12.79) | 2.19 (0.42-11.53) | 1.41 (0.72-2.76) | 1.26 (0.63-2.49) |
| Unknown | 1.01 (0.54-1.90) | 0.99 (0.52-1.88) | 1.63 (0.45-5.91) | 1.63 (0.42-6.31) | 1.14 (0.66-1.98) | 1.07 (0.61-1.88) |
| **Surgery** |  |  |  |  |  |  |
| No | REF | REF | REF | REF | REF | REF |
| Yes | 1.13 (0.75-1.70) | 1.23 (0.75-2.03) | 0.83 (0.42-1.67) | 1.20 (0.50-2.86) | 1.04 (0.74-1.46) | 1.23 (0.81-1.88) |
| **Chemotherapy** |  |  |  |  |  |  |
| No | REF | REF | REF | REF | REF | REF |
| Yes | 1.01 (0.71-1.45) | 1.39 (0.91-2.12) | 1.07 (0.49-2.34) | 1.84 (0.69-4.92) | 1.02 (0.75-1.39) | **1.47 (1.01-2.13)** |
| **Radiotherapy** |  |  |  |  |  |  |
| No | REF | REF | REF | REF | REF | REF |
| Yes | 1.08 (0.80-1.46) | 1.13 (0.82-1.55) | 0.87 (0.45-1.67) | 1.02 (0.49-2.14) | 1.01 (0.78-1.32) | 1.04 (0.79-1.38) |
| **Hormone Therapy** |  |  |  |  |  |  |
| No | REF | REF | REF | REF | REF | REF |
| Yes | 1.41 (0.99-2.00) | **1.47 (1.00-2.15)** | 5.72 (0.46-71.19) | 1.65 (0.07-37.29) | **1.42 (1.01-2.02)** | **1.48 (1.01-2.15)** |
| a. Anxiety is defined as No (did not endorse having symptoms on self-report checklist questionnaire) vs. Yes (endorsed symptoms on checklist); b. Model 1 is adjusted for age at diagnosis, sex (when appropriate), age at diagnosis, time since end of treatment, education level, region, tumor sites, cancer stage, receipt of surgery, receipt of chemotherapy, receipt of radiotherapy, receipt of hormone therapy; c. Model 2 is adjusted for all Model 1 variables in addition to cancer type; d. Odds ratios (OR) and 95% CIs estimated from logistic regression modeling presence of anxiety symptoms (yes vs. no). OR>1 indicates a higher odds of reporting anxiety symptoms for that group in relation to the reference group, OR<1 indicates a lower odds.  *Bolded: Statistically significant at p<0.05* | | | | | | |
